# Prevalence and correlates of leisure-time physical activity among Nigerians

**DOI:** 10.1186/1471-2458-14-529

**Published:** 2014-05-29

**Authors:** Sally N Akarolo-Anthony, Clement A Adebamowo

**Affiliations:** 1 Department of Nutrition, Harvard School of Public Health, 677 Huntington Avenue, Boston MA 02115, USA; 2 Office of Strategic Information and Research, Institute of Human Virology, Abuja, Nigeria; 3 Institute of Human Virology and Greenebaum Cancer Center, University of Maryland School of Medicine, Baltimore MD 20201, USA

**Keywords:** Prevalence, Physical inactivity, Correlates, Nigeria

## Abstract

**Background:**

Physical inactivity levels are rising in many countries with major implications for the prevalence of non-communicable diseases and the general health of the population worldwide. We conducted this study to examine leisure-time physical activity levels among African adults in an urban setting.

**Methods:**

We conducted a cross-sectional study among a random sample of 1,058 adults at a government worksite, in Abuja, an urban Nigerian city. We used log-binomial regression models to estimate the multivariable-adjusted associations of correlates of physical activity.

**Results:**

The mean age of the study population was 42 ± 9.3 years, 60% were men and 40% were women. The mean metabolic equivalent hours per week for all the participants was 6.8 ± 7.2. In univariate analysis comparing the lowest to highest tertiles of physical activity, the prevalence ratio (PR) and (95% confidence interval, CI) was 0.95 (0.81-1.11) *p* = 0.49, comparing women to men; compared to those aged <30 years the PR (95% CI) was 0.70 (0.57-0.86), 0.70 (0.58-0.85) and 0.78 (0.63-0.96) for age 30–39, 40–49 and ≥50 years respectively, *p* for trend = 0.03; compared to those who were normal weight, the PR was 0.93 (0.79-1.10) and 0.90 (0.74-1.09) for overweight and obese persons respectively, *p* = 0.26. The PR for age was attenuated to non-significant levels in multivariable analyses. Being married was a statistically significant correlate of higher physical activity levels, the PR comparing unmarried to married persons in multivariate analysis was 0.81 (0.67-0.97), *p* = 0.03.

**Conclusions:**

More than 80% of urban, professional Nigerian adults do not meet the WHO recommendations of physical activity. Urbanized Africans in this study population had low levels of leisure-time physical activity, independent of age, sex and body-mass index. This has major implications for the prevalence of non-communicable diseases in this population.

## Background

Worldwide, the contribution of different risk factors for disease burden has changed substantially, leading to a shift away from factors associated with high prevalence of communicable diseases which were particularly prevalent in children, towards those for non-communicable diseases that predominate in adulthood [1]. One of these risk factors is physical inactivity which is now the fourth leading risk factor for global morbidity and mortality. Physical inactivity was estimated to be associated with 21 to 25% of breast and colon cancer burden, 27% of diabetes and about 30% of ischemic heart disease burden in 2004 [2]. In 2010, physical inactivity and low physical activity accounted for 3 · 2 million (2 · 7 million to 3 · 7 million) deaths, and 2 · 8% (2 · 4 to 3 · 2%) of Disability-Adjusted Life Years (DALYs) globally [1]. Worldwide, 31% of adults are estimated to be physically inactive and these levels are rising with major public health implications [3]. The WHO recommends adults aged 18 to 64 years should do at least 150 minutes of moderate-intensity or 75 minutes of vigorous-intensity aerobic physical activity, or an equivalent combination of moderate- and vigorous-intensity activity weekly, in order to improve cardiorespiratory and muscular fitness, bone health and reduce the risk of NCDs and depression [4].

It is difficult to estimate trends in physical inactivity in sub-Saharan Africa due to the dearth of data. In a recent systematic review of the prevalence of physical activity in Ghana and Nigeria, 25 to 57% of Nigerians were estimated to be physically inactive though data from the various studies were considered to be limited, poorly reported and not easily comparable [5-9]. Another recent study reported that 68.6% of Nigerian adults living in a metropolitan city in Northern Nigeria were sufficiently active [10]. This wide variation of physical activity prevalence may be due to different socio-demographic characteristics and lifestyle among the groups studied, as well as differences in the definition of physical inactivity and the tools used to measure physical activity.

Objective methods of measuring physical activity, such as accelerometry and heart rate monitoring are considered to be more accurate than self-report methods [11], but as the use of these methods are not feasible in large epidemiology studies involving thousands of participants, physical activity is usually measured by self-reports with questionnaires. A summary of the reliability and criterion validity results for 7 self-report physical activity measures evaluated in adults, reported reliability correlations ranging from 0.34 to 0.89, and criterion validity correlations of 0.14 to 0.53 [12]. The International Physical Activity Questionnaire Short Form (IPAQ-SF), which has been translated to over 20 languages [13], has been used for cross-national assessment of physical activity. A study carried out in 12 countries to determine the reliability and validity of the IPAQ-SF, showed that it had an agreement of about 80% and a correlation coefficient of 0.61 [14]. However, other investigators showed that in this study, the IPAQ-SF overestimated the prevalence of physical inactivity by 50% [15]. In addition, a systematic review showed that the IPAQ-SF typically overestimated physical activity as measured by objective criterion, by an average of 84% [16]. Nevertheless, the IPAQ is still being widely used to measure physical activity variation globally [17-21].

A study to evaluate the reliability and validity of 5 commonly used physical activity questionnaires, showed that the Nurses’ Health Study (NHS) II physical activity questionnaire had statistically significant moderate reproducibility, correlation with average daily pedometer steps, total accelerometer counts per day and cardiovascular fitness [22]. To examine the prevalence and correlates of leisure-time physical activity in Nigeria, we conducted a cross-sectional survey in Abuja, Nigeria’s capital city using the NHS II physical activity questionnaire.

## Methods

### Study population

Between April 2010 and February 2011, we conducted a cross-sectional study among 1,058 individuals at the federal secretariat complex, Abuja, Nigeria, which houses the offices of federal public sector workers in central Nigeria. We generated a list of all offices at the federal secretariat and assigned a random number to each using a random number generator [23]. At each office assigned an odd number, workers and visitors aged over 18 years were approached to participate in the survey. The participation rate was 99% and the participants had a wide range of occupations including skilled labor and professionals. Because it is a federal establishment, the staff distribution is representative of Nigeria’s ethnic and cultural diversity.

### Demographic and socio-economic factors

To verify that we had sampled a diverse population, we collected data on ethnicity, religion, marital status, level of education, and profession. To evaluate socio-economic status (SES), we asked about household possessions including fan, refrigerator, television, bicycle, motorcycle, car, source of drinking water, type of sanitation, type of residence, home ownership, separate room for cooking, source of cooking fuel, respondent self-reported and interviewer-perceived social class [24].

### Physical activity

To assess physical activity levels, interviewers administered the NHS II physical activity questionnaire to the study participants. The questionnaire is a past-year physical activity recall, which measures the average amount of time per week spent on moderate and vigorous leisure time activities, and sedentary activities. Participants reported the average time per week spent on each of the following activities, in the past year: walking, hiking, jogging, running, bicycling, dancing, playing tennis, soccer, squash; golf, swimming, aerobics, weight lifting or resistance exercise. The reproducibility and validity of the NHS physical activity questionnaire has been examined and the correlations between activity reported on questionnaires and that reported on past-week recalls and 7-day diaries were 0.79 and 0.62, respectively [25]. Intensity categories were created by including activities corresponding to the range in metabolic equivalents (METs) for each category (<3 for low, 3–6 for moderate, and >6 for high), based on the recommendation from the Centers for Disease Control and Prevention and the American College of Sports Medicine [26].

### Anthropometric measurements

Trained research nurses measured individual height with a rigid tape measure, in accordance to the World Health Organization (WHO) multinational monitoring of trends and determinants in cardiovascular disease criteria [27]. To measure height, the participants’ were asked to take off his/her shoes, hats or head ties, stand with back to the tape measure, and hold their head in a position where he/she can look straight at a spot, head high, on the opposite wall. A flat rule was placed on the participant’s head, so that the hair (if present) was pressed flat. Height was measured to the nearest centimeter, at the level where the flat rule touched the rigid tape.

To measure weight, participants’ were asked to remove heavy outer garments, empty their pockets and step on a weighing scale, which was placed on a hard, even surface. Weight was estimated using the Omron HBF-510 W Full Body Sensor Body Composition Monitor Scale. Body-mass index categories were defined using the WHO cut points in units of kg/m^2^, normal weight = 18.5 - <25, overweight = 25 - < 30 and obese ≥ 30.

### Statistical analysis

We excluded 17 persons who were underweight, defined as having a body-mass index < 18.5 kg/m^2^. We generated a wealth index using the factor analysis (principal components) procedure and varimax rotation as previously described by Filmer and Pritchett [24], to compute socio-economic status. MET hours were calculated as the product of the duration and frequency of each leisure time physical activity, weighted by an estimate of METs of the activity [28], and summed to give a total activity score in MET hours per week (MET h/wk). Physical activity was analyzed in tertiles, according to the distribution of the study population. Body-mass index was estimated as a ratio of an individual’s weight (kg) and height (m^2^).

We used mean and standard deviation (SD) for descriptive analyses of the continuous variables and *t*-tests to assess the significance of differences between groups in the distribution of continuous variables; *χ*^2^ tests were used for categorical variables. Univariate and multivariate analyses with log-binomial regression models were conducted to examine the associations between potential correlates and the prevalence physical activity, comparing the lowest tertile to the middle and highest tertiles [29-31]. All variables that were associated with physical activity in the univariate analyses with p-value ≤ 0.2, were included in the multivariate model [32]. Prevalence ratios (PRs) and their 95% confidence intervals (CIs) were calculated with the lowest tertile as the reference for all variables. All analyses were conducted with SAS for UNIX statistical software (version 9.2; SAS Institute).

### Ethics

The study was conducted according to the Nigerian National Code for Health Research Ethics and the Declaration of Helsinki. Ethical approval to conduct this study was obtained from the Institute of Human Virology Nigeria Health Research Ethics Committee. Individuals were informed about the study and were requested to consent before participating in the study.

## Results

**Figure 1:**
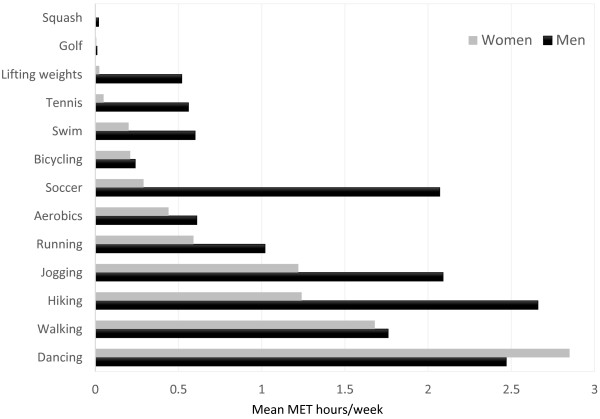
Mean MET hours/week for leisure-time physical activity, by sex.

The mean age (SD) of the participants was 41.6 (9.3) years; 40% (416/1041) were women and 60% (625/1041) were men. Majority of the study population were Christians, married, completed university education and had professional jobs. The mean (SD) MET hours/week (h/wk) for all the participants was 6.8 (7.2) and it was not significantly different for women 6.5 (6.9) compared to men 6.9 (7.4), *p*-value = 0.12. The mean (SD) MET h/wk were 0.9 (0.7), 5.2 (1.7) and 11.5 (6.7) among those in the lowest, middle and highest tertiles of physical activity respectively, *p*-value = <0.001. The mean (SD) BMI of all the participants was 26.8 (7.2); it was significantly different for women, 29 (5.4), compared to men 26 (4.6), *p*-value = <0.001. Most women (74%) and men (56%) in this study were either overweight or obese. The mean (SD) BMI of the participants in the lowest tertile of physical activity was 27.4 (4.8), 27.3 (5.0) for those in the middle tertile and 26.7 (4.6) for those in the highest tertile of physical activity *p*-value = 0.62, suggesting that physical activity was not a major correlate of BMI in this population. Table 1 shows the characteristics of this population overall and by tertiles of MET h/wk.Few of the participants (4%, 40/1041) spent 150 minutes or more on moderate-intensity physical activity per week, 28% (11/40) of these were women and 72% (29/40) were men. Some 13% (139/1041) of the participants spent 75 minutes or more on vigorous-intensity physical activity per week, 26% (36/139) of these were women and 73% (103/139) men. Walking was the most common leisure-time physical activity engaged in by this population. Figure 1 shows the average MET hours/week from different leisure-time physical activities.

**Table 1:** Characteristics of the study population by tertiles of MET-hours* per week (MET- h/wk)

		**Tertile 1**	**Tertile 2**	**Tertile 3**
**Characteristics**	**Overall**	**<2**	**2 - 8**	**>8**
		**n = 334**	**n = 355**	**n = 352**
	Mean ± SD			
**Age (years)**	41.6 ± 9.3	41.5 ± 8.3	42.9 ± 9.2	40.2 ± 10.3
**Physical activity (METs/week)**	6.8 ± 7.2	0.9 ± 0.7	5.2 ± 1.7	15.5 ± 6.7
**Body-mass index (kg/m**^**2**^**)**	26.8 ± 7.2	27.4 ± 4.8	27.3 ± 5.0	26.7 ± 4.6
	Percentages			
**Age categories (years)**				
- <30	11	8	9	17
- 30 – 39	28	31	27	26
- 40 – 49	40	42	41	37
- ≥ 50	21	19	23	20
**Sex**				
- Male	60	63	52	66
- Female	40	37	48	34
**Body-mass index**				
- Normal weight	36	33	38	37
- Overweight	38	40	36	39
- Obese	26	27	26	24
**Religion**				
- Christianity	80	71	85	84
- Islam	20	29	15	16
**Marital status**				
- Married	77	83	76	71
- Not married	23	17	24	29
**Education**				
- None	0.4	0	1	0
- Primary (Elementary school)	0.6	2	0	0
- Secondary (High school)	22	16	24	25
- ≥ Tertiary (College)	77	82	75	75
**Occupation**				
- Self-employed	3	3	2	2
- Unskilled manual	7	9	6	6
- Skilled manual	40	38	42	41
- Professional/executive	50	50	50	51
**Socio-economic status**				
- Low	40	37	41	41
- Middle	40	39	41	41
- High	20	24	18	18
**Smoking**				
- Non-smoker	96	97	98	95
- Current smoker	4	3	2	5
**Alcohol**				
- None	65	71	67	57
- 1 unit/day	12	9	13	15
- 2–5 units/day	21	17	19	25
- 5+ units/day	2	3	1	3

*Metabolic equivalent (MET)-hours is sum of the average time per week spent in each leisure-time physical activity multiplied by the MET value for each activity.

In analysis comparing extreme tertiles, age, religion and marital status were significantly associated with levels of physical activity. Unmarried individuals and those aged less than 30 years were more likely to engage in vigorous physical activity, compared to older persons. These results are shown in Table 2. In multivariate analysis, comparing the lowest (<2 MET h/wk) to highest tertiles (>8 MET h/wk) of physical activity, being married or older than 30 years were associated with higher levels of leisure time physical activity.

**Table 2:** Correlates of the lowest vs. highest tertile of physical activity

**Characteristics**	**N**	**Univariate**		**Multivariate**	
		**PR (95% CI)**	**p-value**	**PR (95% CI)**	**p-value**
**Age categories (years)**			0.03		0.64
- <30	85	1.00		1.00	
- 30 – 39	195	0.70 (0.57, 0.86)		0.86 (0.68, 1.08)	
- 40 – 49	270	0.70 (0.58, 0.85)		0.87 (0.69, 1.11)	
- ≥ 50	136	0.78 (0.63, 0.96)		0.92 (0.72, 1.17)	
**Marital status**			0.0001		0.03
- Not married	156	1.00		1.00	
- Married	530	0.73 (0.63, 0.85)		0.81 (0.67, 0.97)	
**Religion**			<.0001		0.0002
- Christianity	533	1.00			
- Islam	153	0.65 (0.52, 0.81)		0.68 (0.54, 0.85)	
**Socio-economic status**			0.14		0.23
- Low	268	1.00		1.00	
- Middle	273	0.97 (0.83, 1.13)		1.00 (0.86, 1.17)	
- High	145	0.82 (0.66, 1.01)		0.85 (0.70, 1.05)	
**Sex**			0.49		
- Male	441	1.00			
- Female	243	0.95 (0.81, 1.11)			
**Body-mass index (kg/m**^**2**^**)**			0.26		
- Normal weight	242	1.00			
- Overweight	271	0.93 (0.79, 1.10)			
- Obese	173	0.90 (0.74, 1.09)			
**Occupation**			0.32		
- Skilled manual	272	1.00			
- Self-employed	18	0.94 (0.58, 1.51)			
- Unskilled manual	51	0.74 (0.51, 1.05)			
- Professional/executive	345	0.97 (0.83, 1.13)			
**Alcohol**			0.48		
- None	432	1.00			
- 1 unit/day	83	1.09 (0.90, 1.31)			
- 2–5 units/day	146	1.16 (0.75, 1.80)			
- 5+ units/day	19	2.20 (1.99, 2.44)			

PR = Prevalence ratio; CI = Confidence interval.

## Discussion

In this cross-sectional study of physical activity among urbanized adult Nigerians, we found that 4% of the participants spent at least 150 minutes on moderate-intensity physical activity; while 13% spent at least 75 minutes on vigorous-intensity physical activity. To improve cardiorespiratory and muscular fitness, bone health and reduce the risk of NCDs and depression, the WHO recommends adults aged 18 to 64 years should do at least 150 minutes of moderate-intensity or 75 minutes of vigorous-intensity aerobic physical activity, or an equivalent combination of moderate- and vigorous-intensity activity weekly [4].

Although physical inactivity is common in high and low income countries, its prevalence varies worldwide [3,33]. Contrary to expectations that physical inactivity is more common in countries of high income because of the level of development than in those of low income, we found up to two-thirds of our study population did not engage in significant leisure-time physical activity [3], where physical inactivity was defined as not meeting any of three criteria: 30 minutes of moderate-intensity physical activity on at least 5 days every week, 20 minutes of vigorous-intensity physical activity on at least 3 days every week, or an equivalent combination achieving 600 metabolic equivalent minutes per week [4,34]. We also found a high prevalence of overweight and obesity, 74% of the women and 56% of the men were either overweight or obese.

Our results are consistent with other studies in this environment which also found that age, sex, and ethnic origin were associated with physical activity [35]. Previous studies reported that 25–57% of Nigerians were physically inactive [5-10]. However, the methods used in these studies differ from ours. These studies estimated physical activity as significant physical activity at least once a week [9]; or determined from occupational and leisure physical activity [6-8]; or used the IPAQ-SF [10]. A systematic review showed that the IPAQ-SF typically overestimated physical activity as measured by objective criterion, by an average of 84% [16]. Nevertheless, the IPAQ is still being widely used to measure physical activity variation globally [17-21].

Walking was the most common physical activity reported by participants. Walking is a common, accessible, inexpensive form of physical activity and is an important component of total physical activity in adult populations [36]. The comprehensive and neighborhood planning of Abuja facilitates walking and may contribute to the high prevalence of this activity in this study population. Although several studies have shown that neighborhood safety [37], walkability [38], traffic speed and volume [39], and residential density were associated with walking and other forms of physical activity [40], these have not been studied in Abuja. A recent study based on a population in Northern Nigeria showed that perceived safety from crime and traffic were inversely associated with physical activity among Nigerian adults [41].

Dancing was also a commonly reported form of physical activity, especially among women. Given that women are more likely to attend church compared to men [42,43] and the high proportion of Christians in this population, it is likely that most of the weekly dancing occurs at churches. Studies on church based interventions to increase physical activity among African American women in the United States yielded variable results [44]. Interventions to improve physical activities should consider organizations that serve as foci of community engagement similar to the role of churches among African American populations.

A systematic review showed that adults’ leisure-time physical activity, including sports participation, has increased in five high-income countries in the past 20–30 years [45]. In the study population, men were more likely to participate in soccer, tennis and swimming, compared to women. This suggests that women return home and engage in household chores, while men are more likely to participate in sports or engage in physical activity at health clubs [46]. Efforts to promote leisure time physical activity among populations like urbanized Nigerians may build on activities that are already prevalent in the community like walking and dancing instead of introducing new modalities.

Our study is limited by its cross-sectional design and focus on adults in an urban population, thus the results do not account for leisure-time physical activity among children or individuals in rural populations. However, we objectively measured the characteristics of the study population using standardized guidelines and techniques and measured physical activity using validated tools.

## Conclusions

In this population, the proportion of people who engage in leisure time physical activity is low, this has major implications for the prevalence of NCDs in this population. More studies investigating factors associated with physical activity, prevalent and preferred types of physical activities, and intervention studies to increase it should be implemented. Results from such studies may help promote participation in physical activity and support policy choices that can effectively prevent NCDs in African populations.

## Abbreviations

BMI: Body mass index; CI: Confidence interval; DALY: Disability-adjusted life years; IPAQ: International physical activity questionnaire; IPAQ-SF: International physical activity questionnaire short form; MET: Metabolic equivalent; NCD: Non communicable diseases; NHS: Nurses’ health study; PR: Prevalence ratios; SD: Standard deviation.

## Competing interests

The authors’ have no competing interests.

## Authors’ contributions

SNA designed the study, analyzed the data and drafted the manuscript. CAA obtained funds, contributed to the study design and provided critical revisions of the manuscript. Both authors contributed to the study design, read and approved the final manuscript.

## Pre-publication history

The pre-publication history for this paper can be accessed here:

http://www.biomedcentral.com/1471-2458/14/529/prepub
